# Cervical sporotrichosis simulating squamous cell carcinoma in a patient with photodamage^☆^

**DOI:** 10.1016/j.abd.2021.12.009

**Published:** 2022-11-22

**Authors:** Emilly Neves Souza, Lucia Martins Diniz, Luana Amaral de Moura, Valentina Lourenço Lacerda de Oliveira, Henrique Vivacqua Leal Teixeira de Siqueira

**Affiliations:** a Hospital Universitário Cassiano Antônio Moraes, Vitória, ES, Brazil; b Universidade Federal do Espírito Santo, Vitória, ES, Brazil; c Faculdade de Medicina de Jundiaí, Jundiaí, SP, Brazil

Dear Editor,

A 61-year-old female agricultural worker, who kept domestic animals – dogs and cats without diseases – came to be assessed. She was previously hypertensive and used losartan 50 mg/day. She reported the appearance of an erythematous lesion on the cervical region approximately four months after trauma caused by a tree branch. The lesion had grown over a three-month period, with pruritus and local pain, when she came for the appointment. On examination, a plaque with an erythematous-infiltrated border was identified, showing a vegetating and hyperkeratotic center, covered by pustules, meliceric crusts, and black dots, on the anterior cervical region (Figs. 1A and 1B). Skin photodamage was observed around this lesion, with melanosis, leukoderma and solar elastosis. After the diagnostic hypothesis of squamous cell carcinoma (SCC), an incisional biopsy of the lesion was performed. Histopathology showed pseudocarcinomatous hyperplasia with epithelial abscesses, epithelioid granulomatous reaction and mixed inflammatory infiltrate, containing many plasma cells. Direct screening for fungi and alcohol-acid resistant bacilli was negative. The culture in modified Sabouraud agar showed a black filamentous colony with a white halo (Fig. 2), a microculture characteristic of *Sporothrix spp*.

**Figure 1:**
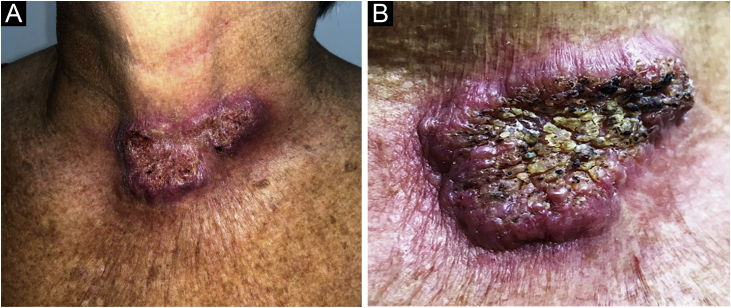
(A) Erythematous-infiltrated plaque with hyperkeratotic surface on the anterior cervical region. (B) Details of the erythematous-infiltrated plaque, showing pustules, meliceric crusts and black dots.

**Figure 2:**
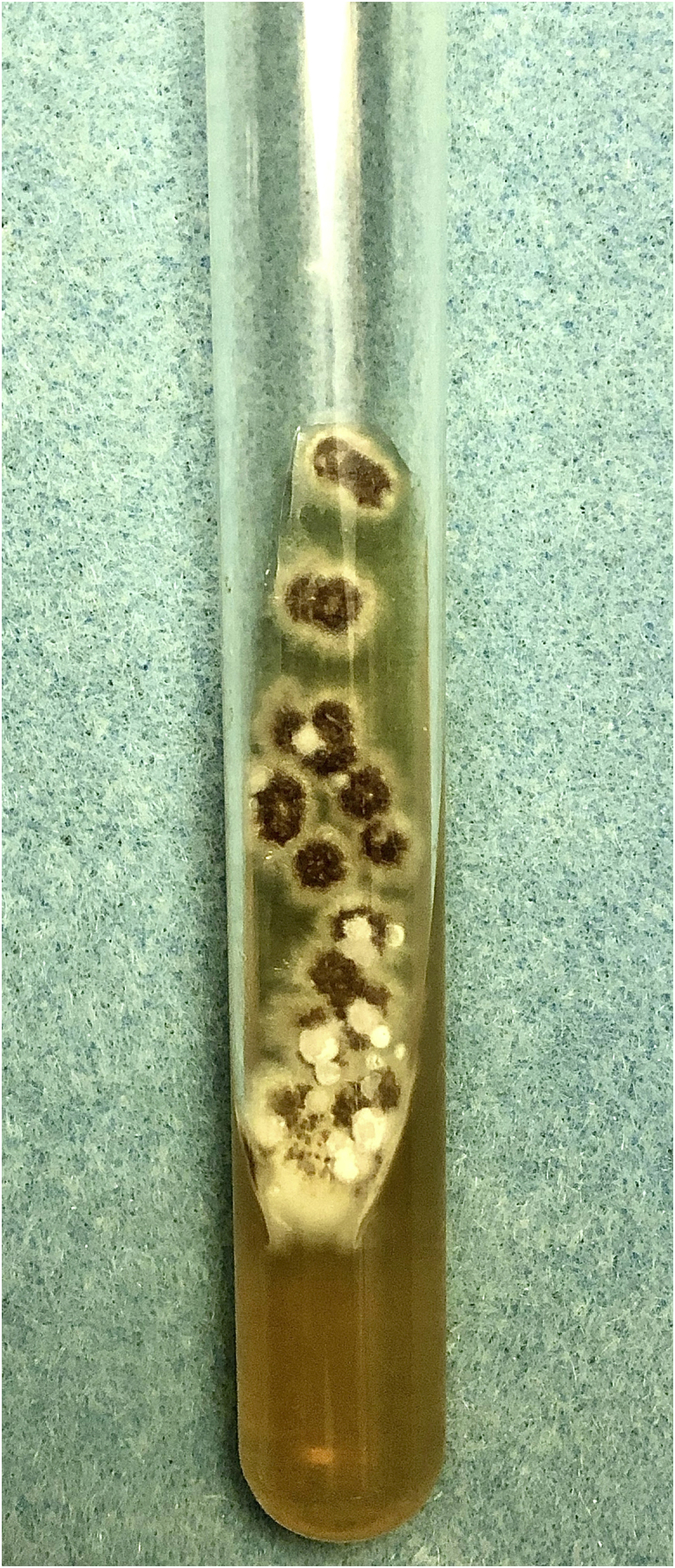
Culture on modified Sabouraud agar medium with macromorphology showing a black filamentous colony with a white halo.

Oral itraconazole 200 mg/day was started and continued for two months, without clinical improvement, being replaced by potassium iodide solution 5 g/day for six months, totaling eight months of treatment. Due to the persistence of an unaesthetic scar (Fig. 3A), four cryosurgery sessions were performed, with satisfactory results (Fig. 3B).

**Figure 3:**
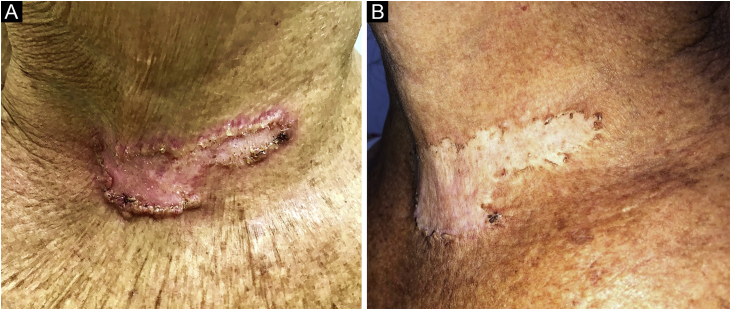
(A) Unaesthetic cicatricial plaque with hyperkeratotic edges after treatment for sporotrichosis. (B) Residual lesion after four cryotherapy sessions.

Sporotrichosis represents the most common subcutaneous mycosis in Latin America,^1^, ^2^ being caused by *Sporothrix spp*., mainly by *S. schenckii*. However, in the last 20 years, *S. brasiliensis* infections have been increasingly reported.^3^ Traditionally, the infection was acquired by cutaneous inoculation of the pathogen into the body extremities after trauma, handling of soil, plants or contaminated organic material. Therefore, agriculture, mining, and floriculture were associated with a higher risk of infection, with a predominance of the lymphocutaneous clinical form in men (80%–95%).^2^, ^3^ However, a change occurred in the transmission profile in the late 1990s, being reported in the urban environment due to contact with infected cats, resulting in an increase in the number of cases in women and children, with atypical cutaneous locations.^4^, ^5^ In Brazil, this has become the most often described form of contamination in recent years.^3^, ^5^ This case report describes risk factors for both forms of contamination.

The localized cutaneous variant, as described in this case, is less frequent and manifests as a single papulonodular lesion, which may develop an infiltrated or vegetating appearance.^6^ The main differential diagnoses include paracoccidioidomycosis, leishmaniasis, chromomycosis, cutaneous tuberculosis, SCC, and non-infectious ulcers.^2^ There are reports in the literature of sporotrichosis simulating keratoacanthoma and Merkel cell carcinoma^7^, ^8^ but no cases mimicking SCC.

The diagnosis can be confirmed by fungal culture and microculture,^2^ as in the present report. The molecular identification of the pathogen was not performed due to the unavailability of such test. The standard treatment comprises oral itraconazole (first choice), potassium iodide solution, or terbinafine.^5^ Treatment duration varies according to the clinical form, fungal virulence and/or the host’s immune status. Cryosurgery and electrosurgery can be combined with medication to reduce treatment duration, also being options for hyperkeratotic lesions.^5^, ^9^ In the present case report, we initially chose the standard treatment with systemic medication, complemented by cryosurgery, due to the size and location of the lesion.

## Financial support

None declared.

## Authors' contributions

Emily Neves Souza: design and planning of the study; drafting and editing of the manuscript; collection, analysis, and interpretation of data; critical review of the literature; critical review of the manuscript.

Lucia Martins Diniz: design and planning of the study; effective participation in research orientation; intellectual participation in the propaedeutic and/or therapeutic conduct of the studied case; critical review of the literature; critical review of the manuscript; approval of the final version of the manuscript.

Luana Amaral de Moura: design and planning of the study; drafting and editing of the manuscript; collection, analysis, and interpretation of data; critical review of the literature.

Valentina Lourenço Lacerda de Oliveira: design and planning of the study; drafting and editing of the manuscript; collection, analysis, and interpretation of data; critical review of the literature.

Henrique Vivacqua Leal Teixeira de Siqueira: design and planning of the study; drafting and editing of the manuscript; collection, analysis, and interpretation of data; critical review of the literature.

## Conflicts of interest

None declared.
